# Policy Considerations for Geriatric Social Workers in Healthcare Settings During COVID-19

**DOI:** 10.1093/geroni/igab046.1473

**Published:** 2021-12-17

**Authors:** Tiffany Washington, Terri Lewinson

**Affiliations:** 1 University of Georgia, Athens, Georgia, United States; 2 The Dartmouth Institute for Health Policy and Clinical Practice, Geisel School of Medicine, Lebanon, New Hampshire, United States

## Abstract

Social workers are essential to the delivery of health care with older adults the during COVID-19 pandemic. This paper focuses on the impact of policies in health care systems that affect geriatric social work practice. Semi-structured interviews were conducted with 55 social workers from a variety of health care settings. Data were analyzed to identify the scope of social work practice in health care settings during the COVID-19 pandemic, and how policies in their respective settings impacted their work. Conditions that impeded participants’ ability to provide quality care and work within their scope of practice included inconsistent expectations of interdisciplinary team members, disparate access to resources, restriction of opportunities to address emotional distress experienced by workers. Recommendations for policy enhancements in health care settings include interprofessional education on effective team communication, protocol development for the equitable distribution of resources among essential workers, and trauma-informed in-service trainings for health care administrators.

